# An Overview of Spinal Injuries due to Dive or Fall into Shallow Water: Our Long-Term, Double-Center Experience from the Aegean Coast

**DOI:** 10.1155/2021/9937730

**Published:** 2021-06-03

**Authors:** Murat Yılmaz, Ersin Ikizoglu, Mert Arslan, Erkin Ozgiray, Kadri Emre Calıskan, Resat Serhat Erbayraktar

**Affiliations:** ^1^Dokuz Eylul University, Faculty of Medicine, Department of Neurosurgery, Izmir, Turkey; ^2^Ege University, Faculty of Medicine, Department of Neurosurgery, Izmir, Turkey

## Abstract

**Purpose:**

We aimed to evaluate the demographic and clinical features of patients with cervical spinal injuries secondary to shallow-water diving and share our therapeutic outcomes.

**Methods:**

A retrospective study was carried out using data extracted from the medical files of 39 patients (3 females and 36 males) who were treated surgically (*n* = 29) or conservatively (*n* = 10). Demographics, clinical features, operative data, American Spine Injury Association (ASIA) impairment scales, and Karnofsky Performance Status (KPS) results were noted.

**Results:**

The average age of our series (*n* = 39) was 31.59 ± 14.80 (range, 14 to 92) years. The vast majority of patients (*n* = 34, 87.2%) presented with isolated cervical trauma. At initial admission, neurological deficits were diagnosed in 22 (56.4%) patients. A single-level cervical involvement was noted in 18 (46.2%) patients, while 21 cases (53.8%) displayed injury involving multiple levels. The levels of cervical injury were C5 (*n* = 16, 41%), C6 (*n* = 11, 28.2%), C7 (*n* = 6, 15.4%), C1 (*n* = 5, 12.8%), and C4 (*n* = 1, 2.6%). A total of 22 patients had neurological deficits at admission. Surgery was performed using anterior (*n* = 21, 72.4%), posterior (*n* = 7, 24.1%), and combined anterior and posterior (*n* = 1, 3.4%) routes. Nine patients (23.1%) exhibited improvement in their neurological deficits. There were significant improvements in both the ASIA impairment scale and KPS results after treatment.

**Conclusion:**

Our data indicated that dive- or fall-related cervical spinal injuries are associated with profound morbidity. Reinforcement of primary prevention, identification of target population, and increased awareness on this topic are the key steps to minimize the frequency and severity of complications and to optimize therapeutic outcomes.

## 1. Introduction

Shallow water is defined as water with a maximum depth of 1.5 m [1]. Shallow-water injuries due to dive or fall into shallow water have catastrophic neurological sequelae in the younger population, at enormous personal cost and a socioeconomic burden to society, medical service providers, and social support structures. The management necessitates intensive resources in the acute and rehabilitation phases. Dive- or fall-related cervical injuries, often leading to quadriplegia and death, constitute a subgroup of cervical spine injuries, particularly due to compression and hyperflexion [2, 3].

Diving injuries occur as a consequence of aquatic recreational activities, and they are one of the leading causes of devastating trauma affecting the spinal column [3, 4]. The spinal column and, particularly, the spinal cord are injured after diving into shallow water. Even though the entire spine is vulnerable, the most commonly affected segment is the cervical spinal area [3–6]. Following dives into shallow pools or sea, the head strikes and stops suddenly which leads to load all of the bodyweight and adversely affects the spine [4, 7]. Thus, the severity of the injury is closely related to the weight of the patient and the height of the jumping place, as well as the depth of the water [8]. This type of injury is more likely to occur for becoming cooler or recreational swimming activity by an unexperienced diver into water or swimming pools with unpredictable depth. Usually, compression-flexion or compression-hyperflexion types of fractures occur [4, 7]. The most important form of spinal damage is compression of the cord attributed to the disruption of the integrity of the cervical column [3, 4]. The incidence of spinal cord injuries (SCI) following dive or fall into shallow ranges between 1.2 and 21% [5, 9, 10]. These injuries exist mainly in the young, healthy, and male population [5, 10–12]. Even though the majority of such spinal injuries are cervical vertebral fractures and dislocations, thoracolumbar injuries, including upper lumbar vertebral fracture, may also be detected [6]. It has been speculated that the actual incidence of dive- or fall-related spinal injuries may be underestimated since some victims may be reported as death due to drowning in the absence of postmortem investigations [7].

We aimed to present our experience with cervical spinal injury due to dive and fall into shallow-water injuries. The clinical and radiological features of these injuries are described and our long-term therapeutic outcomes with current surgical techniques throughout a long-term follow-up are presented.

## 2. Materials and Methods

This retrospective study was performed after the approval of the local institutional review board (2020/15-54). Data derived from the medical records of 39 patients were reviewed concerning their specific conditions. Patients suffering from a spinal injury due to dive or fall into shallow water were included, while patients with minor trauma and/or with vertebral contusions without cervical fracture were excluded. The information was gathered from the databases of 2 university hospitals. These patients were either operated or conservatively treated in the neurosurgery departments of these two tertiary-care centers between January 2012 and September 2019.

Our series consisted of 36 (92.3%) males and 3 (7.7%) females. The average age was 31.59 ± 14.80 years (range: 14 to 92). Data collection involved a review of demographic, personal, clinical, and surgical information, as well as postoperative status, for every patient. The Karnofsky Performance Status (KPS) and American Spine Injury Association (ASIA) scores were recorded before treatment, after surgery, and at the final visit to assess the neurological and functional status of the patients [13–15]. For every patient, sex, age, time (months) of spinal injury, level of the injury, ASIA impairment scale at admission, and in the sixth month, intensity changes in spinal cord magnetic resonance imaging (MRI) findings, spinal treatment options, neurological outcomes, and complications were recorded.

### 2.1. Management of Cervical Trauma

**Figure 1:**
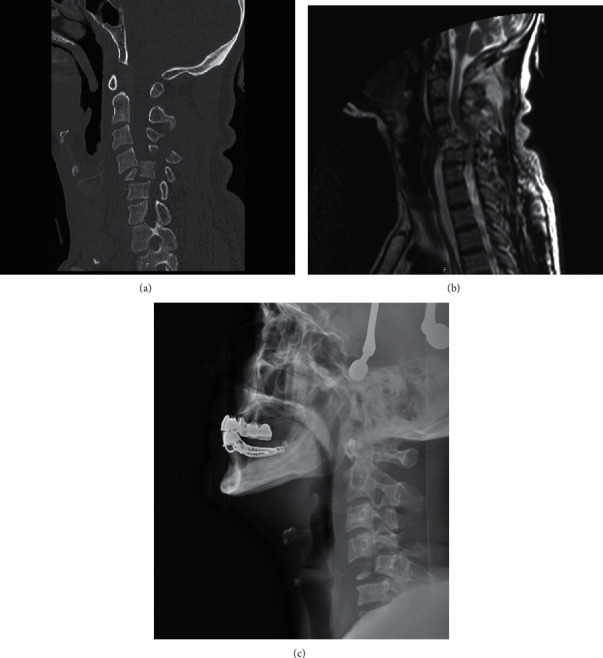
Computerized tomography (a), magnetic resonance imaging (b), and plain radiograph views (c) of cervical spine injury after dive into shallow water.

**Figure 2:**
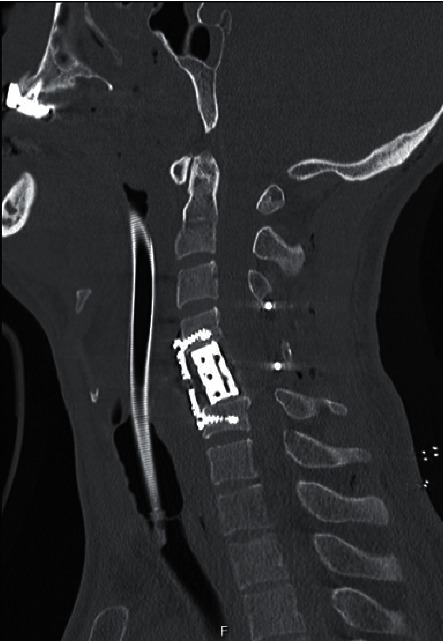
Computerized tomography image after surgical treatment by means of an anterior approach.

**Figure 3:**
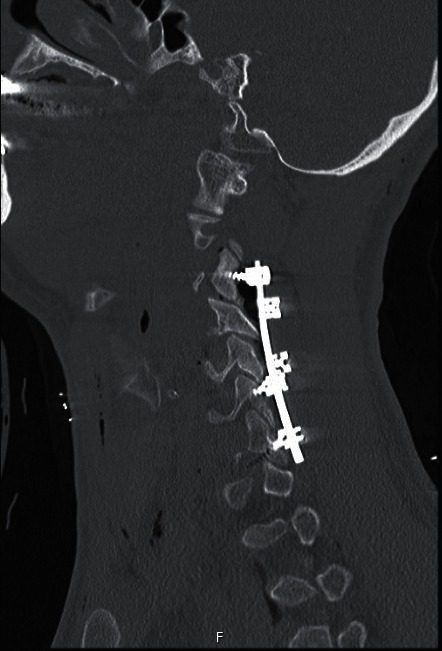
Computerized tomography image after surgical treatment via a posterior approach.

**Figure 4:**
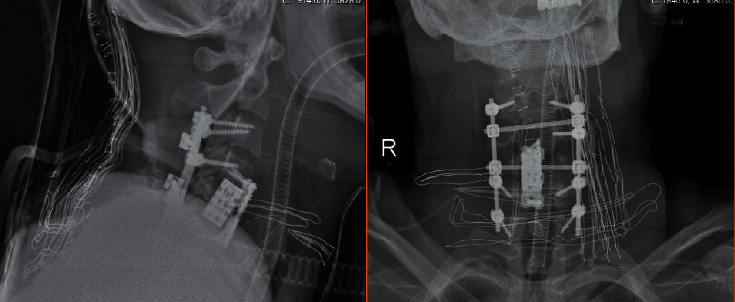
Plain radiograph obtained after surgical repair of cervical injury by a combined anterior and posterior approach.

The patients who admit to the emergency department initially with proven or suspected cervical spinal trauma underwent a cervical radiograph and computerized tomography (CT) scan (Figures 1(a) and 1(b)). In case of any abnormality on neurological examination, MRI was carried out (Figure 1(c)) and the patient was transferred to the intensive-care unit (ICU). Patients with potentially unstable cervical spine or incomplete neurological injury received surgical treatment utilizing anterior, posterior, or combined anterior and posterior approaches (Figures 2–4). Surgical intervention was performed for 29 patients, while 10 patients received conservative medical care. Before surgery, cervical traction with a Gardner-Wells tongs screw was used for eight patients. A reduction was achieved for five patients. Anterior decompression and interbody fusion with cervical corpectomy or discectomy cages and plates were performed for 29 patients (74.3%). Surgical procedures included single or multilevel corpectomy and usual discectomy with screw-plate fixation. In 7 cases, posterior cervical stabilization was performed with the screw-rod construct. Conservative treatment was applied if the spine was stable or in the absence of incomplete neurological injury. Following discharge for the neurosurgery department, a long-term follow-up that involves both clinical and radiological examination (including dynamic imaging of the cervical spine in flexion and extension) was employed.

Spinal injuries were classified either as complete or incomplete as for the status of the lowest sacral segment. The term ‘complete' indicated no motor or sensory function in that segment, while ‘incomplete' as sacral sparing being present. The neurological level was described as the most caudal segment with 5/5 power and normal sensation in both modalities of pinprick and light touch [2].

## 3. Results

In this retrospective study, data collected from the medical files of 39 patients (3 females, 7.7%; 36 males, 92.3%) were analyzed. Patients were treated either surgically (*n* = 29, 74.4%) or conservatively (*n* = 10, 25.6%) The average age of our series (*n* = 39) was 31.59 ± 14.80 (range: 14.00–92.00). In this series, 6 patients (15.4%) were children and adolescents (<18 years of age), 31 (79.5%) were adults (aged between 18 and 50 years), and 2 (5.1%) were elderly (age ≥50).

Comorbidities were detected in 9 (23.1%) patients. These systemic diseases included diabetes mellitus (*n* = 5, 12.8%), hypertension (*n* = 2, 5.1%), hypothyroidism (*n* = 1, 2.6%), and cerebrovascular occlusion (*n* = 1, 2.6%). One patient (2.6%) reported regular use of antiaggregant drugs. The average time of admission to the emergency unit after trauma was 6.3 hours. The vast majority of patients (*n* = 34, 87.2%) presented with isolated cervical trauma, whereas 5 patients (12.8%) had concomitant extracervical injuries. These injuries involved the head (*n* = 2, 5.1%), chest (*n* = 1, 2.6%), extracervical spine (*n* = 1, 2.6%), and extremity bones (*n* = 1, 2.6%). At initial admission, neurological deficits were diagnosed in 22 (56.4%) patients, and 21 of these patients (95.4%) were recruited for surgical treatment.

At initial admission, no cases were diagnosed with neurogenic shock and neither intubation nor ICU stay was needed in any patients. Cervical vertebral computerized tomography scans were routinely obtained from all patients on their initial admission to the emergency unit. Cervical MRI was performed in 35 (89.7%) patients. Of 29 patients who were treated surgically, 28 (96.6%) had undergone preoperative MRI scanning.

Analysis of radiological images yielded a single-level cervical involvement in 18 (46.2%) patients, while 21 cases (53.8%) displayed injury involving multiple levels. The fractures were apparently detected in 38 (97.4%) patients, and these fractures seemed to affect all 3 columns simultaneously and at similar rates. Data as for the type and features of injury are demonstrated in Table 1. Traumatic cervical dislocation and spinal canal compression were noted in 21 (53.8%) and 23 (59.0%) patients, respectively.

Analysis of MRI views indicated that traumatic cervical discopathy was evident in 11 (28.2%) patients and spinal cord edema was determined in 11 (28.2%) cases. Anterior and posterior longitudinal ligament injuries were noted in 14 (35.9%) and 18 (46.2%) patients, respectively. The levels of cervical injury were C5 (*n* = 16, 41%), C6 (*n* = 11, 28.2%), C7 (*n* = 6, 15.4%), C1 (*n* = 5, 12.8%), and C4 (*n* = 1, 2.6%).

A total of 22 patients had presented with neurological deficits. Steroid treatment was started in 21 (53.8%) patients after admission to the emergency unit. Eighteen of these patients had neurological deficits on physical examination.

Traction was carried out in 3 patients, and its average duration until surgery was 33.3 hours. Operative data including surgical techniques are shown in Table 2. Surgery was performed in 29 (74.4%) patients, and the average interval between trauma and surgery was 22.6 hours. Twenty-one (72.4%) of 29 patients scheduled for surgery revealed neurological deficits preoperatively. Surgical interventions were implemented through anterior (*n* = 21, 72.4%), posterior (*n* = 7, 24.1%), and combined anterior and posterior (*n* = 1, 3.4%) routes. Corpectomy was conducted in 22 (75.9%) patients using a corpectomy cage and anterior plate system. In 7 patients (24.1%), laminectomy was carried out using posterior instrumentation. Iliac crest autograft was utilized in only 1 (3.4%) patient. Perioperative dural tear was detected in 7 (24.1%) cases, and neuromonitoring was used during operation in 4 patients (13.8%). Thirteen patients were hospitalized in the ICU postoperatively (44.8%), and the average duration of stay in the ICU was 24.4 days. A tracheotomy was performed in 7 patients who needed to stay in the ICU (53.8%). Postoperative steroid treatment was instructed to 25 of cases treated surgically (86.2%). The average duration of steroid treatment was 208.7 hours.

An overview of ASIA impairment scales and KPS results before treatment, at discharge, and on the final control visit are presented in Tables 3 and 4. In the follow-up period, 9 patients (23.1%) exhibited improvement in their neurological deficits. In 10 patients who received conservative management, neurological findings remained the same. No deterioration was noted in the neurological profiles of any patients in this series. Two patients (6.9%) displayed infection at the site of surgery. The average duration of hospitalization and long-term follow-up were 64.4 and 142 days, respectively. There were significant improvements in both ASIA and KPS results after treatment. After discharge from the hospital, 5 patients demonstrated neurological improvement. Totally, 14 patients displayed neurological improvement compared to their conditions at initial admissions to the emergency unit. Mortality due to cardiac disease occurred in 1 patient (2.6%) on the 6^th^ postoperative day.

## 4. Discussion

The prevalence of cervical spinal injuries increases, particularly, in summer months due to injuries attributed to fall or dive into shallow water. These traumas are not only associated with significant morbidity and mortality, but many patients may have consequential and permanent disabilities [6, 16, 17]. Diving accidents constitute 1.2–22% of all spinal injuries, and 2.5% of all cervical spinal trauma was linked with these accidents [2, 9, 17–19]. In relevant publications, most patients were reported to be young males [2, 9, 18], and our data are in conjunction with current literature. The actual number of cervical injuries due to fall or dive into shallow water may be underestimated, since minor injuries may not always be referred to the tertiary-care centers [5].

Cervical injuries linked with diving occur mainly in the swimming pools and, especially, during summer. Misinterpretation of the depth of water, careless behavior, and alcohol consumption are risk factors for these accidents [11, 18].

The subaxial cervical trauma mechanisms can be classified as compressive flexion, vertical compression, distractive flexion, compressive extension, distractive extension, and lateral extension [20]. Ull et al. stated that the spinal injury may occur in the forms of a fracture without luxation, a fracture with luxation, and a luxation without fracture of the cervical vertebrae [21].

Remarkably, associated injuries are relatively uncommon in diving accidents [5, 9, 11], and our findings are in parallel with these data. Cervical spine injuries mostly occur due to striking the head onto the bottom of the pool or the sea [5]. The most common mechanism is flexion with or without axial compression [5]. Following the strike of the head, the massive force acting on the neck in flexion may lead to subluxation, fracture, and unilateral or bilateral facet dislocations of the cervical vertebrae. Borius et al. reported that a complete neurological injury was the most frequent type, particularly in patients with burst fractures and dislocations [5]. In the emergency unit setting, a CT scan was the main imaging modality to assess spine fractures and plan the treatment policy, whereas MRI was indicated when a neurological deficit was determined. The surgical technique may depend on the preference of the surgeon, and as in our series, the cervical anterior approach is the most common route [4, 5, 22]. No association was demonstrated between fracture types and treatment methods [23]. The main aims were stabilization of the spinal column, prevention of further injury, facilitation of early mobilization, and more rapid rehabilitation [4, 5].

Previous studies demonstrated a relationship between ASIA scale and the socioprofessional outcome [10, 11, 18]. Severe adverse outcomes such as job losses, breakdown of relationships and marriages, psychological problems of the patient and family, intense involvement of hospitalization and rehabilitation processes, costs for continuous care, and loss of productivity are associated with cervical spine injury due to fall or dive into shallow water [4, 5].

Establishment of effective preventive measures such as education and increased awareness for dangers of negligence and reckless behavior are critical to minimize morbidity and complications associated with these accidents [4, 5, 23, 24]. Alcohol consumption must be avoided during swimming and diving and the water must be clear to recognize the depth and identify any immersed objects.

At the beginning of every summer, these preventive efforts must be employed to attenuate the frequency and severity of these injuries. Popularization and reinforcement of the use of warning signs, employment of lifeguards on duty, organization of educational programs at schools, and media campaigns can be functional to achieve these goals [5, 25].

Vlok et al. reported that the most common orthopedic level of injury was C5, and the most frequent associated neurological level of C4 confirmed the severity of these injuries [2]. On the other hand, Aito et al. suggested that the most common neurological level was C6, reminding that more severe injuries may have occurred at this site [9].

Korres et al. reported that concomitant injuries and hospital complications were few but serious [4]. The conservative treatment was justified in selected patients and could lead to improvement of the initial neurological deficit. However, recovery was closely related to the severity of the initial neurological injury. The indications for surgical treatment are posttraumatic instability and persistent neurological deficit. Despite progress in the treatment and rehabilitation of patients suffering from diving injuries, the outcomes are still unsatisfactory. Efforts must be spent to educate young swimmers for the prevention of such catastrophic injuries.

Dive- or fall-related injuries may not always occur in an identical pattern due to the differences in the injury mechanism and the body alignment during trauma. This point must be considered during interpretation of our data.

Our data indicated that patients with cervical spine trauma due to dive or fall into shallow water need to be evaluated rapidly and the diagnostic and therapeutic road map must be tailored carefully and on an individualized basis for every patient. Neurological examination together with a meticulous analysis of clinical and radiological data may provide useful clues for the selection of a suitable strategy.

The main limitations of our study involve retrospective design, and data confined to the experiences of only 2 institutions. Dive- or fall-related injuries may not always occur in an identical pattern due to the differences in the injury mechanism and the body alignment during trauma. Our results must be interpreted cautiously, and extrapolation of our data to larger populations must be carried out carefully.

## 5. Conclusions

In conclusion, spinal injuries associated with fall or dive accidents are linked with substantial morbidity. Neurological consequences are dependent on the efficacy and development of effective treatment strategies. Reinforcement of primary prevention, identification of target population, and increased awareness on this topic are the key steps to minimize the frequency and severity of complications and to optimize therapeutic outcomes. Further trials must be implemented to determine risk factors for dive or fall injuries and to develop more effective preventive strategies.

## Figures and Tables

**Table 1:** The features of spinal cord injury at the cervical region due to dive or fall into shallow water.

Type and site of injury	*n* (%)
Dislocation	21 (53.8)
Cord compression	23 (59)
Traumatic disc injury	11 (28.2)
Preoperative spinal cord edema	23 (59)
Anterior ligament injury	14 (35.9)
Posterior ligament injury	18 (46.2)
Level of lesion	
C1	5 (12.8)
C2	1 (2.6)
C5	16 (41)
C6	11 (28.2)
C7	6 (15.4)
Site of injury (column)	
Anterior	2 (5.1)
Middle	2 (5.1)
Posterior	9 (23.1)
Anterior and middle	8 (20.5)
Middle and posterior	2 (5.1)
Anterior and posterior	2 (85.1)
Anterior, middle, and posterior	13 (33.3)
Missing data	1 (2.6)

**Table 2:** An overview of procedural and perioperative parameters in our population.

Perioperative variable	*n* (%)
Use of neuromonitoring	4 (13.8)
Procedure	
Corpectomy	22 (75.9)
Laminectomy	7 (24.1)
Discectomy	23 (79.3)
Perioperative dural tear	7 (24.1)
Type of surgery	
Anterior	21 (72.4)
Posterior	7 (24.1)
Anterior and posterior	1 (3.4)
Type of operative instrumentation	
Cage and anterior plate	21 (72.4)
Lateral mass	6 (20.7)
Autograft	1 (3.4)
Cage, anterior plate, and transpedicular	1 (3.4)

**Table 3:** The American Spine Injury Association (ASIA) impairment scale profiles in our series.

Variable	ASIA		
	Initially	At discharge	Last control
A	10 (25.6%)	5 (12.8%)	5 (12.8%)
B	5 (12.8%)	6 (15.4%)	4 (10.3%)
C	4 (10.3%)	6 (15.4%)	7 (17.9%)
D	3 (7.7%)	4 (10.3%)	2 (5.1%)
E	17 (43.6%)	17 (43.6%)	20 (51.3%)
Unknown	—	1 (2.6%)	1 (2.6%)

**Table 4:** The Karnofsky Performance Status of our series.

Variable	Karnofsky performance status		
	Initially	At discharge	Last control
20.00	13 (33.3%)	—	—
30.00	5 (12.8%)	4 (10.3%)	1 (2.6%)
40.00	1 (2.6%)	13 (33.3%)	14 (35.9%)
50.00	—	1 (2.6%)	2 (5.1%)
60.00	—	—	1 (2.6%)
80.00	5 (12.8%)	2 (5.1%)	—
90.00	14 (35.9%)	15 (38.5%)	9 (23.1%)
100.00	1 (2.6%)	3 (7.7%)	11 (28.2%)
Unknown	—	1 (2.6%)	1 (2.6%)

## Data Availability

The nature of the data is based on the patient charts and hospital's PACKs. Therefore, all information is available in hospital records. If requested, all information can be forwarded.
